# Citrullination in the pathology of inflammatory and autoimmune disorders: recent advances and future perspectives

**DOI:** 10.1007/s00018-022-04126-3

**Published:** 2022-01-25

**Authors:** Oskar Ciesielski, Marta Biesiekierska, Baptiste Panthu, Mirosław Soszyński, Luciano Pirola, Aneta Balcerczyk

**Affiliations:** 1 Department of Molecular Biophysics, Faculty of Biology and Environmental Protection, University of Lodz, Pomorska 141/143, 90-236 Lodz, Poland; 2 The Bio-Med-Chem Doctoral School, University of Lodz and Lodz Institutes of the Polish Academy of Sciences, University of Lodz, Banacha 12/16, 90-237 Lodz, Poland; 3 CarMeN Laboratory, INSERM Unit 1060, Lyon 1 University, 165 Chemin du Grand Revoyet, BP12, 69495 Pierre Bénite Cedex, France

**Keywords:** Immune disorders, COVID-19, Cancer, Peptidylarginine deiminases (PADs), Anti-citrullinated proteins antibodies (ACPAs), Neutrophil extracellular traps (NETs)

## Abstract

Numerous post-translational modifications (PTMs) govern the collective metabolism of a cell through altering the structure and functions of proteins. The action of the most prevalent PTMs, encompassing phosphorylation, methylation, acylations, ubiquitination and glycosylation is well documented. A less explored protein PTM, conversion of peptidylarginine to citrulline, is the subject of this review. The process of citrullination is catalysed by peptidylarginine deiminases (PADs), a family of conserved enzymes expressed in a variety of human tissues. Accumulating evidence suggest that citrullination plays a significant role in regulating cellular metabolism and gene expression by affecting a multitude of pathways and modulating the chromatin status. Here, we will discuss the biochemical nature of arginine citrullination, the enzymatic machinery behind it and also provide information on the pathological consequences of citrullination in the development of inflammatory diseases (rheumatoid arthritis, multiple sclerosis, psoriasis, systemic lupus erythematosus, periodontitis and COVID-19), cancer and thromboembolism. Finally, developments on inhibitors against protein citrullination and recent clinical trials providing a promising therapeutic approach to inflammatory disease by targeting citrullination are discussed.

## Introduction

Post-translational modifications of histone and non-histone proteins include covalent changes taking place on the amino acid side chain leading to the addition of a chemical group, such as acetyl, methyl, phosphoryl, glycosyl, which significantly change the properties of a protein, its conformation, localization and stability, and help to diversify the protein’s functionalities [1, 2]. In addition to the above-mentioned protein PTMs, particular attention has been given to peptidyl-arginine citrullination, that was found to be an important factor in regulation of the gene expression machinery and, consequently, a regulator of many physiological processes including skin keratinisation, plasticity of the central nervous system or regulation of the immune reactions, by affecting neutrophil extracellular traps (NETs) formation [3, 4]. Citrullination has been also identified as a potential therapeutic target, as conversion of peptidyl-arginine to peptidyl-citrulline plays an important role in promoting tumorigenesis and autoimmunity [5–7]. Recent studies have also shown an association between protein arginine citrullination and COVID-19 severity [8–10].

This review presents the recent advances in the field of protein citrullination and the role of this modification in immune disorders including rheumatoid arthritis, multiple sclerosis, psoriasis, systemic lupus erythematosus, periodontitis and COVID-19. We survey the current-state-of-the-art regarding the enzymes catalysing the reaction, as well as their substrates. We review the identified citrullinated peptide epitopes that are proposed as disease markers, specifically recognized in certain human autoimmune disorders, and discuss the diagnostic features and potential therapeutic value of anti-citrullinated protein antibodies based on ongoing clinical trials.

### Methodology of literature search

Literature search strategy: Medline and Scopus databases were surveyed for original articles and literature reviews with the following keywords and terms, used alone or in combination: *citrullination, deimination, citrulline, peptidylarginine deiminase (PAD), autoimmune disorders, rheumatoid arthritis, multiple sclerosis, systemic lupus erythematosus, periodontitis, psoriasis, inflammatory bowel disease, COVID-19, ACPA, NET, NET formation, NET disorders, therapeutic potential, anti-citrullination therapy, PAD inhibitors.* Adequate clinical trials were searched in the *NIH clinical trials register (clinicaltrials.gov)* and in *The European Council EU Clinical Trials Register (*https://www.clinicaltrialsregister.eu).

### Biochemistry of the citrullination process

**Fig. 1:**
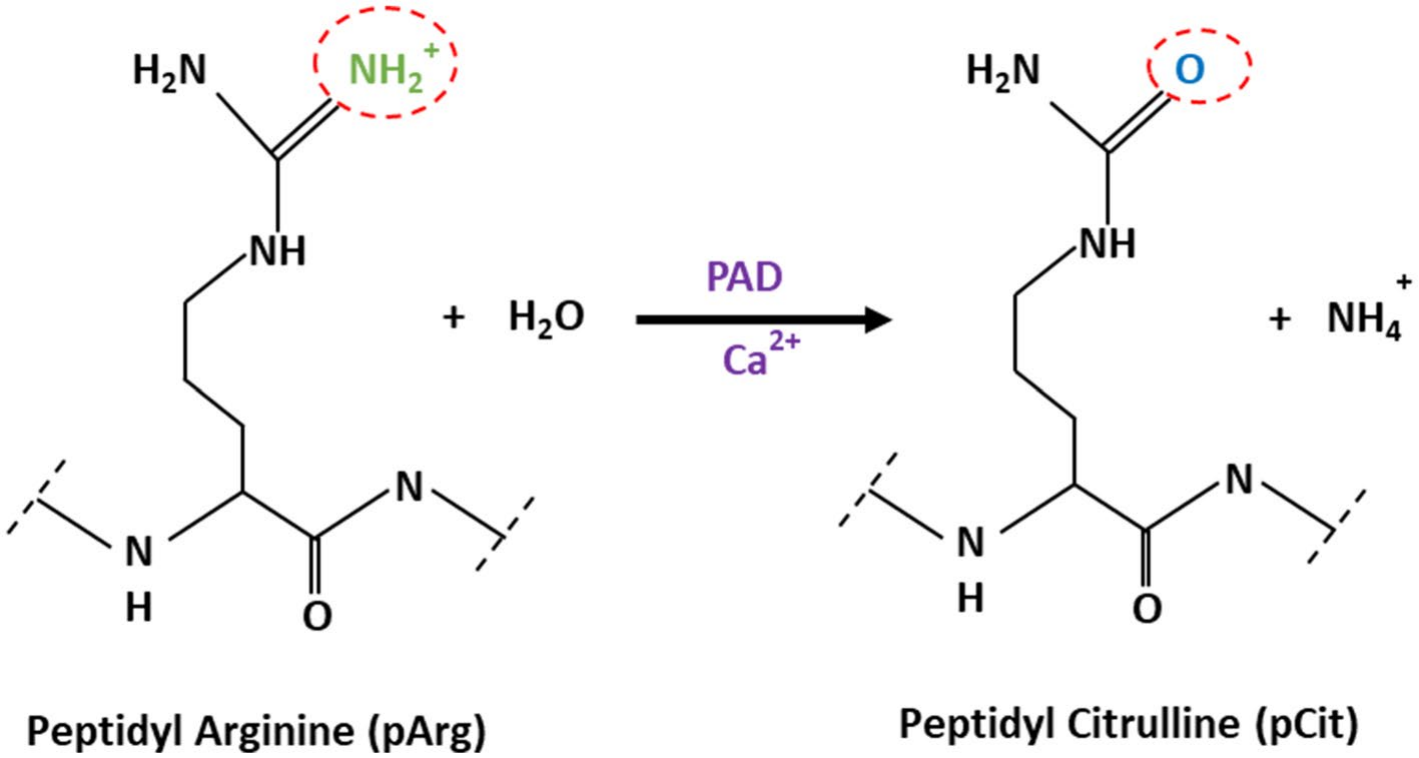
Enzymatic conversion of positively charged arginine into neutral citrulline

Protein citrullination is an irreversible PTM, and refers to the process of conversion of peptidyl-arginine (pArg) to peptidyl-citrulline (pCit). The reaction, known also as deimination, was first described in 1958 by Rogers and Simmonds [11]. As citrulline is not one of the 20 primary amino acids encoded by DNA, pCit only occurs in proteins following pArg deimination. Citrulline is also a metabolite of the urea cycle, the metabolic pathway mediating the detoxification of ammonia into urea, secreted in the urine [12]. Protein citrullination is catalysed by the peptidylarginine deiminase (PAD) enzyme family [13]. The reaction is, at present, considered to be an irreversible process, as no evidence for the existence of a de-citrullination reaction has been reported yet. However, as it was the case with protein methylation, which was initially considered as an irreversible protein PTM prior to the discovery of histone demethylases, a group of decitrullinating enzymes may have yet to be discovered [14]. The chemical mechanism of the deimination reaction is based on a primary ketimine group being replaced by a ketone group, resulting in the loss of the positive charge of arginine and production of ammonia. In contrast to positively charged arginine, citrulline is neutral (Fig. 1) [15].

**Fig. 2:**
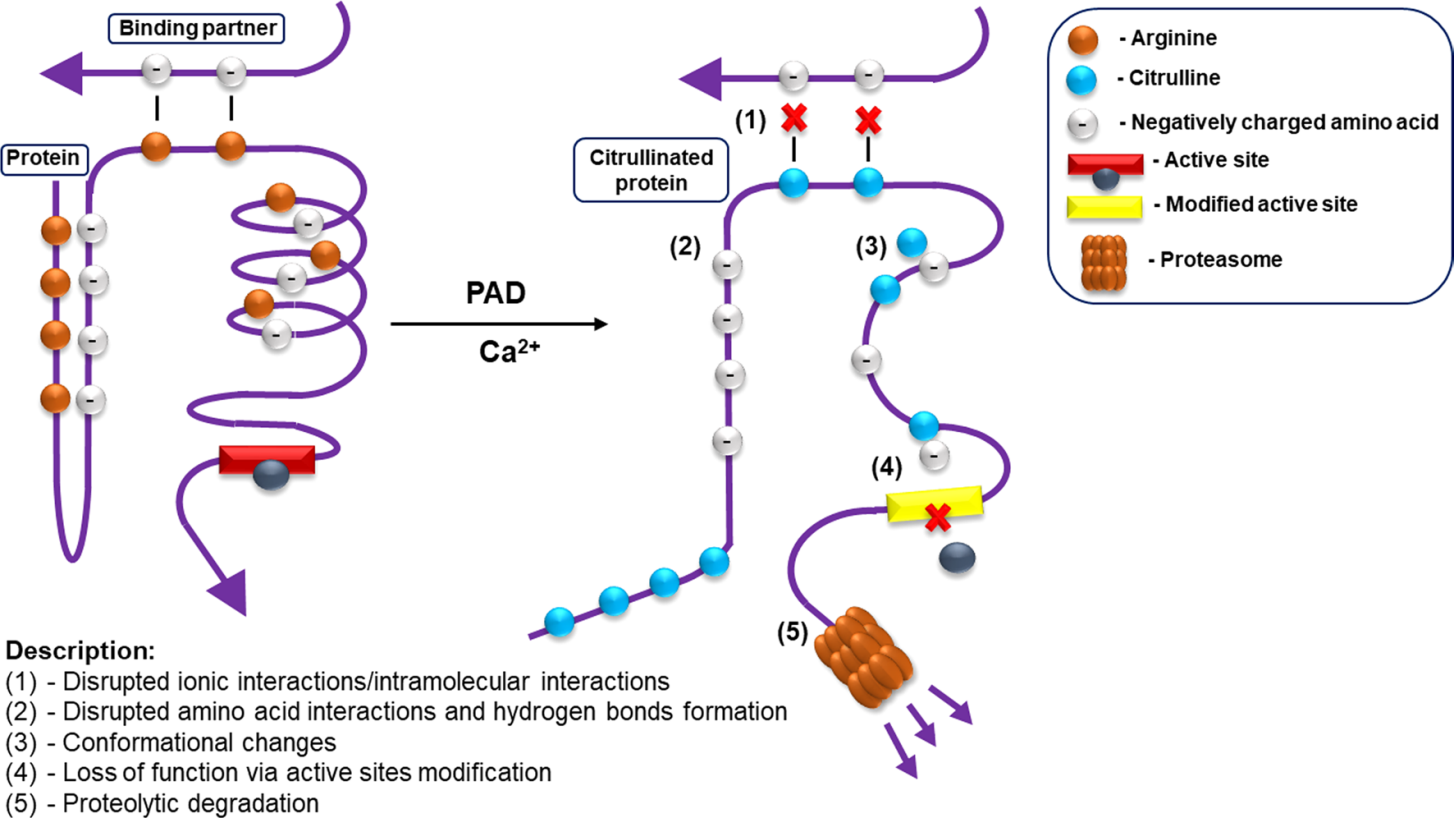
The effects of citrullination on protein–protein interactions

The loss of a positive charge caused by citrullination results in electrostatic and conformational changes of the modified protein, affecting its function by altering binding sites, protein–protein interaction and susceptibility to degradation [15, 16]. Citrullination substantially lowers the amino acid isoelectric point, from 11.41 for arginine to 5.91 for citrulline, thus affecting the acidity of the protein and its potential to form hydrogen bonds and inter amino acid electrostatic interactions (Fig. 2) [17].

The human “citrullinome” includes hundreds of proteins, some of the best known being (i) structural proteins: keratin, fibronectin, actin, tubulin, vimentin and (ii) proteins found in chromatin, notably histones [3]. Recently, protein citrullination has gained much of scientific interest, because of its role in several important biological processes, including skin keratinization, myelin formation and gene expression regulation [4, 5]. One of the best studied processes where citrullination plays a central role is the formation of neutrophil extracellular traps (NETs), the immune system’s first line of defence against infections. While physiological arginine citrullination does occur in the regulation of many physiological pathways, because of its drastic effects on protein–protein interactions and epitope changes, dysregulated arginine citrullination may contribute to the development of severe pathological states [18]. Increased PAD activity, and thus increased protein citrullination, is strongly linked with the occurrence of autoimmune-mediated inflammatory responses such as rheumatoid arthritis (RA) [19]. Moreover, recent reports suggest that excessive protein citrullination may also play a crucial role in non-autoimmune inflammatory diseases such as chronic obstructive pulmonary disease (COPD), or other inflammatory-linked conditions such as: thrombosis, atherosclerosis, psoriasis, cancer and Alzheimer disease [4]. The link between PAD activity and excessive inflammatory response is discussed in detail in the following sections.

### Peptidylarginine deiminases: structure, function and tissue expression

Peptidylarginine deiminases or protein arginine deiminases (PADs), first reported in 1977, belong to the cysteine hydrolases enzyme family (EC 3.5.3.15). PADs drive arginine deimination via a nucleophilic attack of the active site cysteine on the arginine’s guanidinium group, leading to the formation of an intermediate acyl-enzyme which is then hydrolyzed to citrulline [13]. There are five known isotypes of PADs: PAD1, PAD2, PAD3, PAD4 and PAD6, with PAD6 being catalytically inactive, that are distributed in a myriad of human cell types showing a highly tissue-specific expression pattern and consequently perform various functions. All the human PAD isozymes have a length ranging from 663 to 694 amino acids, a molecular weight around 74 kDa and share 70–95% homology within their sequences [13], UniProtKB database]. The overall structure of PADs is composed of two N-terminal immunoglobulin-like domains with regulatory function which are connected to a C-terminal catalytic domain. PAD2 and PAD4 crystallize as homodimers with the two holoenzyme’s active sites exposed on the same face of the dimer [20, 21]. The PADs active site contains a conserved nucleophilic cysteine (C645 in PAD1 and 4; C647 in PAD2; C646 in PAD3), a conserved histidine (H471) and two aspartates (D350 and D473). The cysteine and histidine residues are the two catalytic residues, with the two aspartates acting to stabilize the positively charged peptidyl-arginine substrate between the active site cysteine and histidine. PADs contain 4 (PAD1), 5 (PAD3, 4) and 6 (PAD2) Ca^2+^ binding sites, and these are crucial for PADs catalytic activity, as calcium increases PAD activity by > 10,000 fold. Ca^2+^ binding at these sites leads to a series of conformational changes that induce a catalytically competent active site [22].

Only PAD2 and PAD4 isozymes contain a nuclear localization signal, targeting them to the nucleus where they deiminate histones H1, H3 and H4 [21, 23]. All PAD isotypes require a high concentration of calcium to perform their function, typically above 100 µM. Therefore, in most physiological states, PADs remain inactive. Upon binding of Ca^2+^, PADs undergo a conformational change that moves the cysteine of the active site into a competent position for catalysis [21, 24]. As shown by Tarcsa et al., not all arginine residues within a polypeptide are amenable to citrullination by PADs. In the case of the two proteins: filaggrin and trichohyalin, the secondary structure is a determinant factor for arginine accessibility and potential conversion to citrulline [25]. Arginine residues in filaggrin, a protein rich in β-turn structures, were frequently citrullinated, as opposed to low citrullination observed in trichohyalin, a protein in which single α-helix structures prevail [25]. Arginine residues located in disordered structures rapidly undergo the conversion to citrulline, with efficiency up to 95% [25, 26]. Furthermore, the reaction efficiency also depends on the amino acid sequence which contains the target arginine. Arginines located next to glutamic acid residues or flanked by prolines are reported to be less frequently citrullinated, in contrast to arginines proximal to aspartic acid residues, which are more likely to be a substrate for PADs [25, 27]. However, consensus sequences for arginine to citrulline conversion were reported only in filaggrin and trichohyalin, and no further studies involving other proteins have been published to date. Performing such studies on other targets is warranted as it would clarify whether these sequence-specific citrullination rules generally apply to known PAD targets.

As mentioned above, citrullination is crucial for several physiological processes, each being driven by one or more PAD isotypes. Processes reported to require PAD action include: skin differentiation (PAD1, PAD3), terminal differentiation of keratinocytes (PAD1, PAD3), brain and nervous system functioning (PAD2), immune cells differentiation (PAD2, PAD4), immune response—NET formation (PAD4) and proper functioning of the female reproductive system (PAD6). Table 1 summarises the most recent findings on the physiological roles of PAD isotypes and also presents the identified molecular targets/protein substrates.

**Table 1:** Physiological roles of PADs

PAD isotype	Expression profile	Known substrates	Physiological roles	Refs
PAD1	Immune cells, keratinocytes, hair follicle, epidermis, uterus	Keratin, Filaggrin, S100A3, MEK1-ERK1/2-MMP2 signalling enzymes	Skin differentiation, terminal differentiation of keratinocytes, epithelial-mesenchymal transition	[5, 28–30]
PAD2	Immune cells, spleen, thymus, skeletal muscle, brain, glial cells, colon, kidney, pancreas, breast, epidermal, bone marrow, salivary gland, secretory gland, ear, eye	MPB, CXCL10, CXCL11, Vimentin, Actin, GFAP, S100A3, Histone H3, Histone H4	Oligodendrocyte differentiation and myelination, brain plasticity, female reproduction, transcription regulation	[5, 29, 31–33]
PAD3	Immune cells, keratinocytes, hair follicles, nerves	Filaggrin, Vimentin, Trichohyalin, S100A3	Skin differentiation, hair follicle formation, terminal differentiation of keratinocytes	[5, 28, 29],
PAD4	Immune cells, brain, uterus, bone marrow, joints, cancerous tissues (e.g. breast carcinomas, lung carcinomas)	NFC1, NCF2, S100A3, Collagen Type I, HAT p300, NPM1, GSK3β, ING4, RPS2, FUS, EWS,0 TAF15, ADAMTS13, Histone H1 (R54Cit), Histone H2A (R3Cit), Histone H3 (R17Cit, R26Cit, R2Cit, R8Cit), Histone H4 (R23Cit, R3Cit)	Immune cells differentiation, cellular differentiation, NET formation, gene expression regulation, tumorigenesis	[5, 13, 28, 29, 33–41]
PAD6	Ovary, early embryo, testicles, ovum, oocyte, thymus	α-tubulin	Cytoskeletal reorganization in the egg and early embryo, preimplantation cleavage, early embryonic development, oocyte cytoskeletal sheet formation and female fertility	[42–44]

#### Expression of PADs in immune cells

Different PAD isoforms present tissue-specific expression patterns [45] (Table 1). Due to the key role of PADs in mediating inflammatory responses, the identification of the expression of specific PADs isoforms in immune cells is of particular importance, allowing their identification as potential therapeutic targets. PAD1 and PAD3 gene and protein expression is very low in peripheral blood mononuclear cells (PBMCs), but detected in specific leukocytes populations (Table 2). On the contrary, gene expression of PAD2 and PAD4 was observed in PBMCs, with detectable expression in B cells, T cells, NK cells and monocytes (CD14^+^, CD3^−^, CD19^−^, CD56^−^). During monocyte to macrophage differentiation, PAD4 gene expression is lost, but PAD4 protein expression persists, indicating its stability. In contrast, PAD2 mRNA levels remain unchanged during monocyte to macrophage differentiation, but PAD2 protein is only detected in macrophages, as PAD2 translation is initiated during macrophage differentiation [46]. Expression of PAD4 in monocytes and PAD2/PAD4 in resting macrophages is, however, not associated to constitutive citrullination. A raise in cytosolic calcium concentration, as occurring during NETs formation or apoptosis, leading to an activatory conformational change by the binding of Ca^2+^ to the Ca^2+^ binding sites of PADs is necessary to induce their activation [29].

**Table 2:** Estimated protein expression levels based on HIPED (the Human Integrated Protein Expression Database), a unified database of protein abundance in human tissues, residing within GeneCards

Leukocytes/PAD isotype	PAD1	PAD2	PAD3	PAD4	PAD6
Agranulocytes					
Monocytes	+ +	+ + +	−	+ +	−
B lymphocytes	+ + +	+	−	+	−
T lymphocytes					
CD4	−	−	−	−	−
CD8	−	+	−	+	−
Other	+ +	−	−	−	−
Nk-cells	−	+	−	+	−
Granulocytes					
Neutrophils	−	+ +	+	+ + +	−
Eosinophils	nd	nd	nd	+	nd
Basophils	nd	nd	nd	nd	nd

*nd* no data; − no expression; + low/ +  + moderate/ +  +  + high estimated protein expression level

### PAD inhibitors

The link between PAD activity and inflammatory conditions is strong, with citrullination being causal for the formation of NETs. Therefore, recent years have witnessed an ever growing interest in the development of PAD inhibitors (PADis).

Several reversible wide-spectrum PAD inhibitors have been identified, including streptomycin, minocycline, taxol, chlorotetracycline, ruthenium red and sanguinarine [47–50]. Even more attention has been devoted to the finding of specific PAD4 inhibitors, as PAD4 appears to be the main driver of most citrullination dependent diseases. Initially, GSK121 was identified as a lead compound in a screen for PAD4 inhibitors, and was the basis for lead optimization to produce the more potent and specific PAD4 inhibitors GSK484 and GSK199 [50]. These specific PAD4 inhibitors showed significant inhibition of protein citrullination and NET formation in human and mouse neutrophils [51]. In a recent study, Aliko et al. tested six compounds proposed as novel PAD4 inhibitors, one of which (compound 4—SC97362) showed significant inhibitory potential [52].

Reversible PADis are relatively weak inhibitors, most likely due to the small active site cavity which requires calcium binding to the enzyme to accommodate the side chain of peptidyl-arginine substrate [53]. Therefore, the most potent PADis described to date are compounds that covalently modify the active site cysteine, in an irreversible way [50]. An activity-based protein profiling high-throughput screening platform identified NSC95397 and streptonigrin as irreversible PADis [50]. It was also shown that 2-chloroacetamide covalently binds to and inhibits PAD4 [54–56]. In 2006, Luo et al*.* described two compounds Cl-amidine and F-amidine which act as pan-PAD inhibitors through covalent modification of the active site cysteine in a time and concentration dependent manner [56, 57]. Both inhibitors have shown promising results for potential use as therapeutics. Cl-amidine reduced ex vivo NET formation by preventing histone H3 citrullination [58] and reduced the severity of arthritis by reducing the production of autoantibodies against citrullinated epitopes in a mouse model of collagen-induced arthritis [59]. F-amidine was shown to inhibit the citrullination of p300 by PAD4 [57]. Incorporation of a carboxylate moiety at the *ortho* position of Cl-amidine and F-amidine produced the second generation of PADis, namely *o*-Cl-amidine and *o*-F-amidine [50, 53]. These modified inhibitors show significant improvement of potency and selectivity, and are more potent in inhibiting histone H3 citrullination in HL-60 cells (> 100-fold) [50, 60]. Two more modifications of Cl-amidine and F-amidine were synthesized by introducing an N-terminal biphenyl group and a C-terminal benzimidazole group, namely BB-Cl-amidine and BB-F-amidine. The introduced groups increase the hydrophobicity, bioavailability and stability of the compounds [50]. Although these PADis show similar potency to Cl-amidine and F-amidine, BB-Cl-amidine has a longer half-life in vivo then Cl-amidine, and is also more cytotoxic to U2OS cells (~ 20 fold) [50, 61]. BB-Cl-amidine was shown to inhibit NET formation and to downregulate the expression of type I interferon genes in MRL/lpr mice [61]. Another derivative of Cl-amidine—PAD2-IN-1—is a potent and highly selective PAD2 inhibitor (over 95-fold and 79-fold more potent over PAD4 and PAD3, respectively) [62]. Interestingly, two peptidic PADis with C-terminal chloroacetamidine- or fluoro-conjugated orthenine were identified in a small library: TDFA (Thr–Asp–F-amidine) and TDCA (Thr–Asp–Cl-amidine). These two compounds show high selectivity to specific PAD isoforms: TDFA towards PAD4 and TDCA towards PAD1 and PAD4 [50, 53]. Although numerous PADis have been reported in the literature, as presented in Table 3, Cl-amidine and BB-Cl-amidine remain the most investigated.

**Table 3:** An overview of PAD inhibitors

Type	Inhibitor	IUPAC name (from PubChem)	Inhibitory potential	Refs
Reversible inhibitors	Streptomycin	2-[(1R,2R,3S,4R,5R,6S)-3-(diaminomethylideneamino)-4-[(2R,3R,4R,5S)-3-[(2S,3S,4S,5R,6S)-4,5-dihydroxy-6-(hydroxymethyl)-3-(methylamino)oxan-2-yl]oxy-4-formyl-4-hydroxy-5-methyloxolan-2-yl]oxy-2,5,6-trihydroxycyclohexyl]guanidine	IC_50_ PAD4 = 1.8 mM	[50, 55, 63]
	Minocyline	(4S,4aS,5aR,12aR)-4,7-bis(dimethylamino)-1,10,11,12a-tetrahydroxy-3,12-dioxo-4a,5,5a,6-tetrahydro-4H-tetracene-2-carboxamide	IC_50_ PAD4 = 620 µM	[48, 50, 64]
	Taxol	[4,12-diacetyloxy-15-(3-benzamido-2-hydroxy-3-phenylpropanoyl)oxy-1,9-dihydroxy-10,14,17,17-tetramethyl-11-oxo-6-oxatetracyclo[11.3.1.03,10.04,7]heptadec-13-en-2-yl] benzoate	Pan-PAD inhibition at 12.5 mM	[49, 50, 65, 66]
	Chlortetracycline	(4S,4aS,5aS,6S,12aR)-7-chloro-4-(dimethylamino)-1,6,10,11,12a-pentahydroxy-6-methyl-3,12-dioxo-4,4a,5,5a-tetrahydrotetracene-2-carboxamide	IC_50_ PAD4 = 100 µM	[48, 50, 63, 67]
	Ruthenium red	Azane; ruthenium(2 +); hexachloride; dihydrate	*K*_*i*_ PAD1 = 30 µM *K*_*i*_ PAD2: 17 µM *K*_*i*_ PAD3: 25 µM *K*_*i*_ PAD4: 10 µM	[50]
	Sanguinarine	24-methyl-5,7,18,20-tetraoxa-24-azoniahexacyclo[11.11.0.02,10.04,8.014,22.017,21]tetracosa-1(24),2,4(8),9,11,13,15,17(21),22-nonaene	*K*_*i*_ PAD1 = 2000 µM *K*_*i*_ PAD2 = 100 µM *K*_*i*_ PAD3 = 60 µM *K*_*i*_ PAD4 = 80 µM	[50, 68]
	GSK121	(3-aminopiperidin-1-yl)(1-methyl-2-(1-methyl-1H-indol-2-yl)-1H-benzo[d]imidazol-5-yl)methanone 2,2,2-trifluoroacetate	IC_50_ PAD4 = 3.2 µM	[50, 51]
	GSK484	(3-amino-4-hydroxy-piperidin-1-yl)-(8-(7-(cyclopropyl-methyl)-7-aza-bicyclo[4.3.0]nona-1(6),2,4,8-tetraen-8-yl)-5-methoxy-7-methyl-7,9-diaza-bicyclo[4.3.0]nona-1,3,5,8-tetraen-3-yl)-methanone	IC_50_ PAD4 = 50 nM	[50, 51, 69, 70]
	GSK199	(R)-(3-Aminopiperidin-1-yl)(2-(1-ethyl-1H-pyrrolo[2,3-b]pyridin-2-yl)-7-methoxy-1-methyl-1H-benzo[d]imidazol-5-yl)methanone hydrochloride	IC_50_ PAD4 = 200 nM	[28, 50, 51, 71]
Irreversible inhibitors	NSC95397	2,3-bis(2-hydroxyethylsulfanyl)naphthalene-1,4-dione	*K*_*i*_ PAD1 = 175 M^−1^ min^−1^ *K*_*i*_ PAD2 = 1600 M^−1^ min^−1^ *K*_*i*_ PAD3 = 9150 M^−1^ min^−1^ *K*_*i*_ PAD4 = 4530 M^−1^ min^−1^	[50, 68, 72, 73]
	Streptonigrin	5-amino-6-(7-amino-6-methoxy-5,8-dioxoquinolin-2-yl)-4-(2-hydroxy-3,4-dimethoxyphenyl)-3-methylpyridine-2-carboxylic acid	*K*_*i*_ PAD1 = 3700 M^−1^ min^−1^ *K*_*i*_ PAD2 = 12,000 M^−1^ min^−1^ *K*_*i*_ PAD3 = 3500 M^−1^ min^−1^ *K*_*i*_ PAD4 = 440,000 M^−1^ min^−1^	[50, 74]
	2-chloro-acetamide	2-chloroacetamide	–	[50, 54, 75, 76]
	Cl-amidine	N-[(2S)-1-amino-5-[(1-amino-2-chloroethylidene)amino]-1-oxopentan-2-yl]benzamide	*K*_*i*_ PAD1 = 37,000 M^−1^ min^−1^ *K*_*i*_ PAD2 = 1200 M^−1^ min^−1^ *K*_*i*_ PAD3 = 2000 M^−1^ min^−1^ *K*_*i*_ PAD4 = 13,000 M^−1^ min^−1^	[31. 51, 56, 58, 60, 61 64]
	F-amidine	N-[(2S)-1-amino-5-[(1-amino-2-fluoroethylidene)amino]-1-oxopentan-2-yl]benzamide	*K*_*i*_ PAD1 = 2800 M^−1^ min^−1^ *K*_*i*_ PAD2 = 380 M^−1^ min^−1^ *K*_*i*_ PAD3 = 170 M^−1^ min^−1^ *K*_*i*_ PAD4 = 3000 M^−1^ min^−1^	[50, 54, 56, 62]
	*o*-Cl-amidine	N-α-(2-carboxyl)benzoyl-N5-(2-Chloro-1-iminoethyl)-l-ornithine amide	*K*_*i*_ PAD1 = 100,000 M^−1^ min^−1^ *K*_*i*_ PAD2 = 40,000 M^−1^ min^−1^ *K*_*i*_ PAD3 = 8000 M^−1^ min^−1^ *K*_*i*_ PAD4 = 50,000 M^−1^ min^−1^	[50, 60]
	*o*-F-amidine	N-α-(2-carboxyl)benzoyl-N5-(2-fluoro-1-iminoethyl)-l-ornithine amide	*K*_*i*_ PAD1 = 40,000 M^−1^ min^−1^ *K*_*i*_ PAD2 = 180,000 M^−1^ min^−1^ *K*_*i*_ PAD3 = 7000 M^−1^ min^−1^ *K*_*i*_ PAD4 = 45,000 M^−1^ min^−1^	[50, 60]
	BB-Cl-amidine	N-[(1S)-4-[(1-amino-2-chloroethylidene)amino]-1-(1H-benzimidazol-2-yl)butyl]-4-phenylbenzamide	*K*_*i*_ PAD1 = 16 100 M^−1^ min^−1^ *K*_*i*_ PAD2 = 4 100 M^−1^ min^−1^ *K*_*i*_ PAD3 = 6 800 M^−1^ min^−1^ *K*_*i*_ PAD4 = 13 300 M^−1^ min^−1^ EC_50_ PAD4 = 8.8 µM	[50, 56, 61, 70]
	BB-F-amidine	N-[(1S)-4-[(1-amino-2-fluoroethylidene)amino]-1-(1H-benzimidazol-2-yl)butyl]-4-phenylbenzamide	*K*_*i*_ PAD1 = 900 M^−1^ min^−1^ *K*_*i*_ PAD2 = 1200 M^−1^ min^−1^ *K*_*i*_ PAD3 = 3400 M^−1^ min^−1^ *K*_*i*_ PAD4 = 3750 M^−1^ min^−1^	[50]
	PAD2-IN-1	N-[(1S)-4-[(1-amino-2-fluoroethylidene)amino]-1-(4-ethoxy-1-methylbenzimidazol-2-yl)butyl]-3-oxo-1,2-dihydroisoindole-4-carboxamide	IC_50_ PAD2 = 8.3 µM	[50, 62]
	TDFA	N-Acetyl-l-threonyl-l-α-aspartyl-N5-(2-fluoroethanimidoyl)-l-ornithinamide	*K*_*i*_ PAD4 = 25,000 M^−1^ min^−1^	[50, 77, 78]
	TDCA	N-Acetyl-l-threonyl-l-α-aspartyl-N5-(2-chloroethanimidoyl)-l-ornithinamide	*K*_*i*_ PAD1 = 20,000 M^−1^ min^−1^ *K*_*i*_ PAD4 = 24,000 M^−1^ min^−1^	[50, 78]

## Protein citrullination as a hallmark of immune disorders

Findings of the recent years have permitted to establish that citrullination is a process strongly linked to inflammation [79]. Multiple studies show that most immune disorders are characterized by an excess of citrullinated proteins [19]. The human immune system in normal conditions is not self-reacting to citrullinated proteins. However, as citrullination substantially affects protein structure and folding, some epitopes may undergo changes or new epitopes may be formed upon protein citrullination, which in turn may trigger immune response against citrullinated host protein resulting in autoimmune reaction [80]. Moreover, in most patients with autoimmune-mediated conditions, PAD2 and PAD4 levels are elevated, further indicating a strong correlation between protein citrullination and these diseases [4]. In line with these observations, high levels of citrullinated proteins are often found in patients prior to the onset of rheumatoid arthritis [81].

Abnormal PAD activity is now firmly associated with a number of immune disorders, such as: rheumatoid arthritis, multiple sclerosis, systemic lupus erythematosus, periodontitis, psoriasis and inflammatory bowel disease (Table 4) [15, 82]. In the following sections the latest findings on PAD’s involvement in immune system disorders, including COVID-19, recently affecting the world globally, are discussed.

**Table 4:** Reported involvement of PADs in immune disorders

PAD isotype	Disease	Role in disease/molecular target (if applicable)	Refs
PAD1	Psoriasis	Changes in skin differentiation pathways/keratin	[13, 83]
PAD2	Multiple sclerosis, rheumatoid arthritis, COVID-19	Hypercitrullination of MBP resulting in myelin sheet disruption /MBP	[84, 85]
PAD4	Rheumatoid arthritis, multiple sclerosis, cancer, COVID-19	Hypercitrullination of many proteins e.g. vimentin, filaggrin, MP resulting in production of ACPAs and acute autoinflammatory reactions, excessive NET formation, gene expression regulation via histone citrullination	[5, 13, 31, 86]
PAD6	Infertility	Early embryonic arrest/unknown	[42, 43]

### PAD4-mediated NETs: the double edged sword of the immune system

**Fig. 3:**
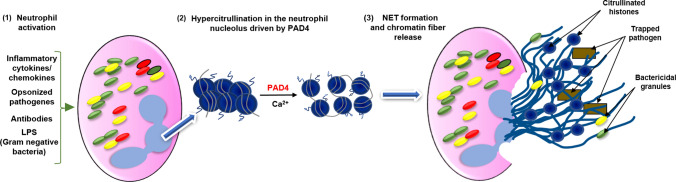
Schematic presentation of the classical concept for PAD 4-mediated NET formation. (1) Neutrophils stimulated by a range of proinflammatory stimuli, including microbial agents, trigger a cascade of reactions that ultimately entrap and kill pathogens. (2) PAD4-driven hypercitrullination of proteins, including histones, causes changes in the chromatin architecture and relaxation of its structure. (3) This allows neutrophils to release chromatin fibres that are able to bind and kill bacteria as well as degrade virulence factors

Neutrophils, a subclass of blood granulocytes, constitute the vanguard of the human innate immune response to invading pathogens: bacteria, fungi and viruses [86, 87]. Their modes of action include: phagocytosis—consisting of the direct engulfing of pathogens, production of reactive oxygen species (ROS) and release of cytokines and chemokines, which recruit other immune cells and enhance the host immune response [88, 89]. In 2004, the formation of neutrophil extracellular traps (NETs) was identified as a new mechanism of neutrophil action against invading pathogens [90]. NETs are composed of a decondensed chromatin scaffold decorated with factors possessing high bactericidal properties such as: citrullinated histones, cathepsin G, lactoferrin, proteinase 3, neutrophil elastase (NE), myeloperoxidase (MPO) and peptidoglycan-binding proteins [91]. NETs have been also reported to be highly effective in fighting viral infections by blocking viral replication through PKC (Protein Kinase C) pathway inhibition [89, 92]. As shown by numerous studies, NET formation is also a citrullination dependent process, mainly driven by PAD4-mediated citrullination of histone H3 [93]. Neutrophils show a high expression level for PAD4, further indicating an important function for this enzyme in cell specific immunoreactivity [94]. The classic NET activation pathway via the induction of the formation of terminal complement complexes (C5b–9) is described in detail in other reviews [95]. Briefly, during NET formation, PAD4 citrullinates histones in neutrophils, resulting in chromatin decondensation due to the loss of histone positive charges [29, 96]. Part of the citrullinated histones may be excised from the chromatic template and subsequently released in the extracellular space where anti-pathogen effects takes place [97]. The mechanism is schematically presented on Fig. 3. The importance of PAD4 action for NETs was initially shown in studies utilizing pan-PAD inhibitors, such as Cl-amidine, that significantly reduces NET formation [29]. Subsequently, novel reversible PAD4-specific inhibitors, belonging to the GSK family (GSK121, GSK199 and GSK484) were shown to be sufficient to disrupt mouse and human NET formation [51]. Several studies suggest also a role for PAD2 in NET action, however, in contrast to PAD4, PAD2 is currently not viewed as a factor required for NET formation, but rather as an intrinsic component of mature NETs [98–100]. Although beneficial during pathogen invasion, citrullination driven NET formation can also have detrimental effects on the host, thus it may be perceived as a double edged sword. Increased action of PAD enzymes in NETs may result in loss of immune tolerance to citrullinated proteins, and recently multiple studies have shown that excessive NETs build up may contribute to the development of several autoimmune and autoinflammatory diseases [91].

### Rheumatoid arthritis

**Fig. 4:**
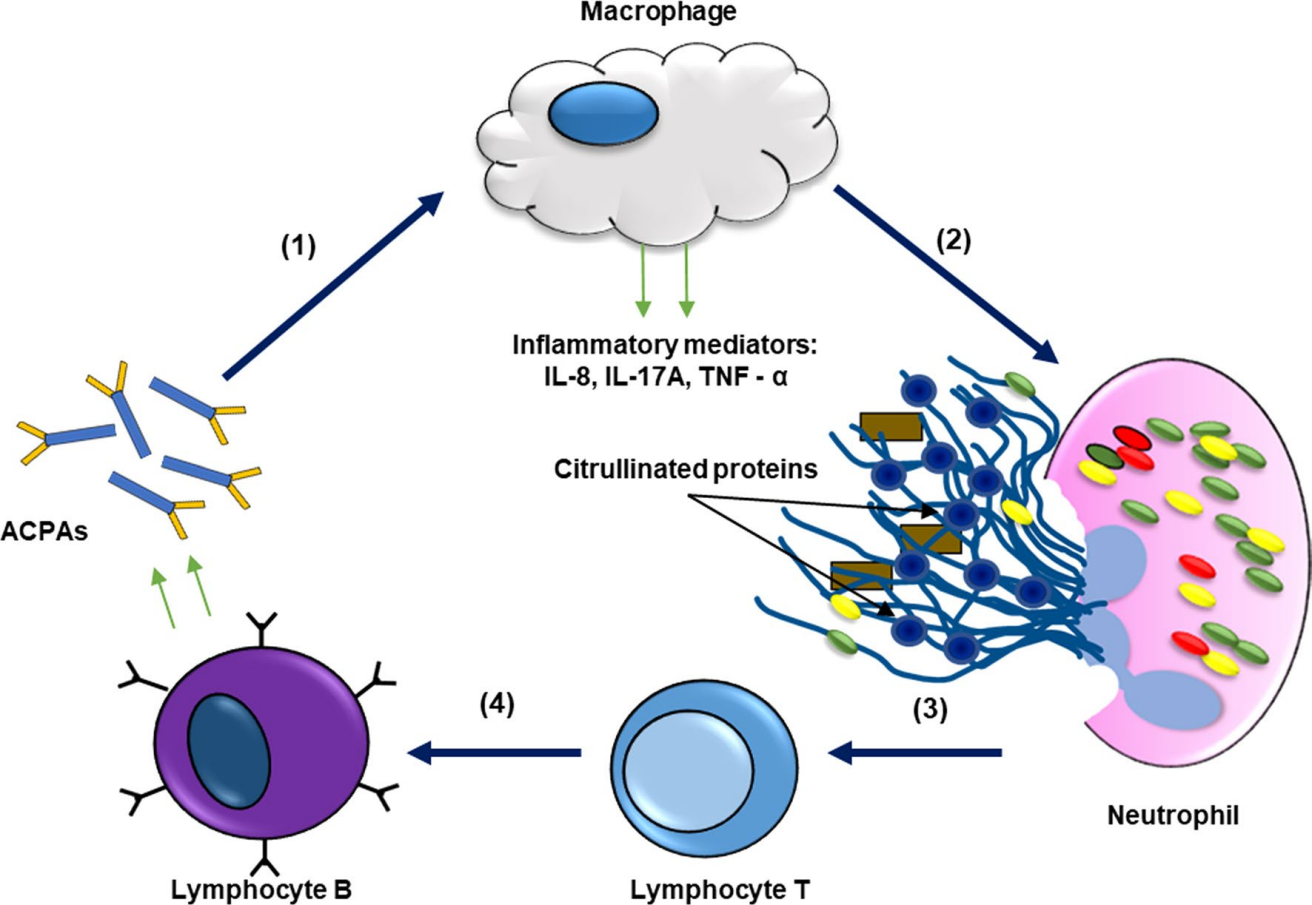
The proposed role of NETs and increasing ACPAs level in rheumatoid arthritis. (1) ACPAs induce the production and release of inflammatory mediators by macrophages. (2) Inflammatory mediators secreted from macrophages induce NET formation. (3) Citrullinated proteins released during NETosis are recognised by lymphocytes T. (4) Antigen presentation and induction of ACPA production resulting in a vicious inflammatory circle with an excessive immune response

Rheumatoid arthritis (RA) is an immune disorder in which protein citrullination plays a prominent role. RA is a chronic and progressive disease, characterized by a symmetric deformity of joints, which may result in structural damage to the joint and systemic inflammation [101]. A specific characteristic of the disease is the presence of high quantities of autoantibodies directed against post-translationally modified proteins in synovial tissue fluids [19]. Pathogenesis of RA can progress for decades, with initial years of asymptomatic autoimmunity, until symptoms, including tissues erosion and joint inflammation become apparent [102]. RA can cause disability as chronic synovial inflammation, as well as hyperplasia, drive joint damaging and bone thinning [103]. As shown by numerous reports, protein citrullination, and thus PAD activity, is one of the main players in RA pathogenesis. In RA patients the expression, activity as well as activation of PAD2 and PAD4 are increased [104]. A model for increased PAD activity in inflamed joints proposed that PAD enzymes originate from monocytes and macrophages recruited to the joints [13, 46]. In this model, macrophages and monocytes rich in PADs are eventually degraded and undergo apoptosis, which causes PAD activation due to rising levels of calcium [46]. These activated PADs are then free to citrullinate both cellular and extracellular proteins, building an excessive pool of citrullinated proteins in the joints that eventually cause the loss of immune tolerance [19]. Some of the best known PAD2 and PAD4 targets in RA are: vimentin, fibrinogen, fibronectin, anti-thrombin and α-enolase [19]. When immune tolerance to citrullinated proteins is lost, anti-citrullinated proteins antibodies (ACPAs) are produced that recognize the newly formed epitopes [105]. ACPAs are detected in 75% of RA patients, and, therefore, their detection is used as a predictive method before disease onset as they are present in the serum of patients even 5 years prior to the symptomatic phase of the disease [3]. Although a large number of proteins are targeted by PADs in RA, only a few were identified as targets for ACPAs namely: vimentin, α-enolase and fibrinogen. These ACPAs demonstrate arthritogenic potential and greatly stimulate the progression of the autoimmune response, suggesting their direct involvement in the pathogenesis of RA. The biological actions of ACPAs include: stimulation/promotion of proinflammatory cytokine production, induction of osteoclastogenesis and stimulation of NET formation [105]. In vitro studies demonstrated that ACPAs from RA patients activate the complement via the classical and alternative pathway, which further exacerbate the autoimmune response in the joints [106]. It was also reported that ACPAs may stimulate macrophages to produce and release inflammatory mediators (e.g. TNF-α) in the joints [107, 108]. NETs are reported to be highly dysfunctional in RA, and serve as a driving force for increasing ACPAs [6]. PAD4-mediated NET formation leads to citrullination of histones H2A, H2B and H4 serving as antigens and, in addition, generation of citrullinated vimentin which stimulates the secretion of proinflammatory cytokines [109, 110]. Increased NET formation may be induced by inflammatory mediators such as: TNF-α, IL-8 and IL-17. These mediators are released by immune cells in large quantities in response to ACPAs and NETs, and amplify the occurrence of ACPAs antigens. Therefore, a cycle of NET formation and ACPAs accumulation can be hypothesized: the initial ACPAs induce inflammatory mediators which in turn activate NET formation and the production of even more ACPAs (Fig. 4) [111]. The vicious circle of reciprocal reinforcement of ACPAs and NETs does not apply, however, to all reported ACPAs. The use of monoclonal therapeutic antibodies designed to target citrullinated N-termini of histones 2A and 4 demonstrated that citrullinated histones are specific targets for therapeutic intervention with anti CitH2/H4 therapeutic antibodies, leading to a decrease in NET formation [111]. The development of therapeutic anti-citrullinated antibodies will be discussed more in detail in the paragraph "Diagnostic and therapeutic potential of citrullination". It was also recently reported that NETs can directly injure cartilage in RA, with PAD2 action directly contributing to this process [112]. To summarize, PAD catalytic activity and protein citrullination have been linked to the pathogenesis of RA for over 20 years, however, the role for NETs in RA etiology and the comprehension of the mechanisms leading to the development of strong autoimmune responses to citrullinated proteins still require further elucidation.

#### N-glycosylation determines the pathogenicity of ACPAs

N-linked glycans play an important role in immunity and, in autoimmune disease, the severity of the response to autoantibodies can be modulated by the degree of glycosylation on the Fc or variable domain (Fab) of immunoglobulins [113]. Several studies demonstrated that glycosylation patterns of ACPAs modulate their pathogenicity and change during autoimmune disease progression. To evaluate the establishment of Fc glycosylation patterns on ACPAs before onset of rheumatoid arthritis and during the development of the disease, serum was collected at baseline from patients with ACPA-positive arthralgia—a joint pain condition predisposing to RA—and at a various follow-up time points. Within this cohort, the 57% of the participants who developed arthritis displayed a higher degree of ACPA-IgG1 Fc fucosylation at baseline and prior to diagnosis as compared to the fucosylation levels observed in total IgG1 [114]. Similarly, a strong enrichment of N-linked glycans on the Fab-domain of ACPAs IgG, leading to a 10–20 kDa higher molecular weight compared to non-autoreactive IgG, was identified in RA patients [115]. Structural analysis indicated that > 90% of ACPA-IgG Fab-domain contains highly sialylated glycans, representing a five-fold enrichment in sialylation as compared to control IgGs from the same donor [116]. High sialylation was more notable in ACPAs from synovial fluids than serum ACPAs from the same patient [116]. These data indicate that ACPAs glycosylation contribute to their arthritogenic potential and increased glycosylation stimulates the progression to RA. The measure of ACPAs glycosylation levels may potentially be used as a biomarker to monitor disease progression and ACPAs IgG glycosylation may be a target for disease treatment.

### Multiple sclerosis

Multiple sclerosis (MS) is an autoimmune disease characterized by demyelination of the central nervous system (CNS) [117]. Demyelination leads to loss of nerve signal transduction, causing severe adverse neurological effects that are clinically manifested as ataxia, weakness, visual loss, movement disorders, bladder dysfunction and even complete disability [118, 119]. The etiology and pathogenesis of MS still remains unclear. It is believed that genetic factors combined with environmental factors and vitamin D deficiency contribute towards a higher risk of MS development [120]. Another theory indicates that epigenetic changes, highly susceptible to environmental factors, passed from parents to progeny may also contribute to MS onset [121]. One of the proposed mechanisms for the occurrence of demyelination and MS development is the failure to maintain the myelin sheet structure due to excessive myelin basic protein (MBP) citrullination. MPB citrullination was shown to be carried out by PAD2, and increased expression of this isotype is reported in the white matter and peripheral blood of MS patients [84, 85]. Overexpression of PAD2 and the associated enhancement of citrullination of MPB in MS patients is the result of hypomethylation of the PAD2 gene promoter [84]. The role of PAD2 in demyelination is also supported by a study in which PAD2 knock-out mice exhibit no CNS citrullination and demyelination [65]. In healthy individuals, 18% of MBP arginines are citrullinated whereas in MS patients the citrullinated arginine to total arginine ratio increases to approximately 45% [4]. The enhanced citrullination profile of MBP causes extensive loss of net positive charges and partial unfolding of MBP molecules. MBP hypercitrullination prevents the transition of lipid bilayers into compact multilayers, resulting in highly unstable myelin sheets and loss of proper nerve signal transduction [65]. To summarise, current evidence indicates that excessive protein citrullination, mediated by PAD2 overexpression, may be an etiologic factor in MS onset.

### Systemic lupus erythematosus

Systemic lupus erythematosus (SLE) is a chronic systemic autoimmune disease that can manifest itself in any part of the body. Due to its heterogeneity in clinical manifestation, SLE diagnosis and treatment can be challenging [122]. SLE is characterized by loss of immune tolerance and abnormal B- and T-cell function, eventually leading to significant morbidity and mortality [122]. A hallmark of SLE is the release of a plethora of autoantibodies, including ACPAs [123]. Tissues of patients affected by SLE exhibit increased levels of citrullinated proteins. PAD4 is directly involved in SLE through its role in NET formation and histone citrullination [124] while the role for PAD2 and other PAD isotypes in SLE has not yet been investigated. The observed high levels of citrullinated proteins contribute to the loss of immune tolerance and high production of ACPAs, which subsequently cause the detrimental autoimmune response in SLE [125].

### Psoriasis

Psoriasis is a relatively common chronic immune-mediated skin disorder, displaying clinical manifestations of well-demarcated red scaly lesions on the skin [125]. Psoriasis lesions are typically found on the scalp, elbow and knees, and the disease may also be accompanied by arthritis as comorbidity [126]. The cause of psoriasis remains to be elucidated. Clinically, psoriasis results from rapid proliferation of keratinocytes in the epidermis’ basal layer and high migration of excess keratinocytes to the skin’s surface. Due to such rapid maturation, keratinocytes accumulate on the skin surface forming thick dry patches [127, 128]. The reason for the hyperproliferation of keratinocytes is unknown, however, the infiltration of immune cells in the skin may play a decisive role in lesion formation [126]. Recently, Benhadou et al*.* showed that the contribution of immune cells, keratinocytes, endothelial cells and several other skin-resident cells is required for the maintenance and progression of psoriatic alterations [129]. There is also evidence supporting a driving role of citrullination in psoriasis. PAD1 is expressed at high level in keratinocytes, where it citrullinates keratin K1 during terminal differentiation [83]. Deimination of arginines in keratin K1 facilitates the compaction of keratin filaments, a phenomenon required for normal epidermis cornification [4]. Several groups reported that in skin samples from psoriasis patients there is loss of citrullinated keratin K1 [83, 126, 130]. Lack of citrullinated keratin K1 results in disturbed epidermis cornification, and may be a contributing factor to psoriasis. It was reported that, besides lowered citrullinated keratin K1 levels in psoriasis patients, psoriatic cornified cell layers displayed lowered levels of global protein citrullination [83]. As expression levels of PADs in psoriatic lesions are not altered, the PADs catalytic activity is enhanced in the course of the disease [130]. Lately, a role for NETs was proposed in psoriasis, but their role is not defined. In psoriatic patients, the amount of NETs formed was increased and correlated with disease severity [125]. The administration of a PAD4 inhibitor Cl-amidine alleviated the gravity of psoriatic lesions in a mouse model and decreased NETs formation, suggesting a role for both NETs and PAD4 activity in this disorder [131].

### Periodontitis

Periodontitis (PD) is an inflammatory disease of the oral cavity affecting the tissues supporting teeth that, if left untreated, may result in teeth loss. PD is caused by the gram-negative anaerobic bacterium *Porphyromonas gingivalis* [4, 132]. The *P. gingivalis* peptidylarginine deiminase enzyme (PPAD) can rapidly citrullinate both bacterial and human host proteins such as fibrinogen and α-enolase [4]. PPAD released from *P. gingivalis* can spread into the host’s connective tissue, where can modify/citrullinate epidermal growth factor (EGF), thus blocking its recognition by the epithelium. This mechanism delays the local healing process and breaks the local protective epithelial cell-periodontal tissue barrier [132]. PPAD also generates a pool of new potent antigenic epitopes that, when present in excess, break the tolerance barrier, which will result in generating ACPAs and may induce an acute autoimmune response [132, 133]. High levels of ACPAs are observed in patients with aggressive form of periodontitis [134]. Analysis of the possible link between PD and RA (as the autoimmune response induced by *P. gingivalis* is similar to that observed during RA) revealed that host and microbial PAD activities are highly elevated in RA and non-RA periodontitis patients. Moreover, the severity of periodontal conditions was increased in patients with RA comorbidity as compared to healthy individuals [133, 135]. Although PD is not an autoimmune disease itself, it may induce a severe autoimmune response of the host, were citrullinated proteins are an important factor for disease development.

### Inflammatory bowel disease

Inflammatory bowel diseases (IBDs) are chronic inflammatory conditions characterized by inflammation of the intestinal mucosa. The two main subtypes of IBD include ulcerative colitis (UC) and Crohn’s disease (CD) [136]. In UC patients, chronic inflammation is limited to the intestinal mucosae and in CD patients the inflammation can be transmural in the whole gastrointestinal tract [136], rendering CD more aggressive. In healthy human colon enzymatic PAD activity is negligible, suggesting that deimination processes are not physiologically required in the intestine. In contrast, PADs and citrullinated protein levels are increased in human UC [79]. As in the case of RA, elevated levels of citrullinated proteins may trigger an atypical immune response to the newly formed epitopes and high levels of ACPAs induce inflammation of the UC [136, 137]. Moreover, immunohistochemical staining of human UC samples demonstrated strong expression of both PAD2 and PAD4, and treatment of UC mice with Cl-amidine reduced inflammation [137]. A cohort study of 114 Japanese patients with UC suggested that specific PAD4 haplotypes previously known to be associated with RA are also found in UC [138]. Recent studies suggest an even more prominent role for PAD4 in IBD, as it was found that IBD occurrence was positively correlated with PAD4 and negatively correlated with PAD2 [82, 139]. Also, some researchers have tried to link IBD to NETs formation. Given the key role of citrullination in this process, higher correlation of increased PAD4 expression with IBD incidence may be explained. Inflamed colon of UC patients displayed increased NET formation and overexpression of NET-associated proteins, including citrullinated histones and PAD4, with both responses being diminished in subjects receiving streptonigrin, a selective PAD4 inhibitor [140, 141]. Similar results were recently reported by Li et al., in which NETs formation increased tissue damage and thrombotic tendency in IBD [142]. In conclusion, targeting NETs in IBD may provide an effective therapeutic approach to alleviate the disease [143].

### COVID-19 and pulmonary diseases

COVID-19 is a novel disease caused by infection with the Severe Acute Respiratory Syndrome coronavirus 2 (SARS-CoV-2) [144]. The disease spread across the world from late 2019 causing a global pandemic and a plethora of global health, sociological and economical adverse effects [145, 146]. The disease can manifest itself with multiple symptoms, making it hard to properly diagnose patients in the absence of a molecular test detecting the viral genome [147, 148]. The most common COVID-19 symptoms include: influenza-like symptoms, diarrhoea, anosomia, osteoarticular disorders and a cytokine storm which affects the functioning of the immune system [149]. The severity of the symptoms varies between patients, ranging from asymptomatic cases, to mild consequences, to lethal respiratory failure [150]. As scientists around the world race to find effective treatments to COVID-19, in addition to currently available preventive vaccination approaches, a link between the disease and protein citrullination starts to emerge. Sera from COVID-19 patients have elevated levels of citrullinated histone 3 as well as myeloperoxidase-DNA complexes, two specific markers of NETs [8]. Increased level of histone H3 citrullination in COVID-positive patients was found to be correlated with increased leukocyte, granulocytes and cytokine IL-8 levels, indicating the occurrence of an acute inflammatory response [9]. What is even more interesting, in the context of arginine deiminiation’s role in determining the severity of COVID-19, is that SARS-CoV-2 positive patients show increased levels of NET formation, and sera from those patients cause NET formation when added to control neutrophils [10, 151]. The lungs are the most devastated organ by COVID-19, and elevated NETs formation was reported in lung epithelial tissue and tracheal aspirate, indicating that NETs may be an important factor to the severity of the disease [151–153]. As PAD4 activity is essential for NET formation, and elevated levels of citrullinated H3 are detected in COVID-19, it may be possible that the virus alters PAD4 activity. A recent study by Arisan et al*.* based on transcriptomic data from patients’ biopsies and in vitro experiments linked PADs activity to SARS-CoV-2 infection [154]. However, more studies on this subject must be performed to unravel the complete picture of PAD involvement, and NET formation, in COVID-19 [154]. Zuo et al. propose that the positive correlation between platelet count and citrullinated H3 levels, and thus the abnormal increased platelet count, is one of the possible explanations for the vascular clinical manifestations of COVID-19 [8, 151]. Interestingly, elevated levels of citrullinated circulating nucleosomes were reported in COVID-19 patients [150]. Cavalier et al*.* propose to use circulating citrullinated nucleosomes as biomarkers for COVID-19, as citrullination levels are positively correlated to disease severity [150]. SARS-CoV-2 is known to cause, in a percentage of patients, a post-acute COVID-19 syndrome, termed long-COVID, characterized by persistent symptoms and delayed long-term complications [155]. Such prolonged effects may result in even more excessive accumulation of citrullinated proteins, and, therefore, lead to loss of immune tolerance. A first case of increased levels of ACPA in a post-COVID-19 patient showing initial symptoms of RA was recently reported [156]. This may be the first of many reported post-COVID-19 symptoms related to citrullination. These recent data linking citrullination and NETs to COVID-19 indicate that citrullination events may be highly relevant to the physiophathology of COVID-19 and more generally in pulmonary diseases associated with lung inflammation.

One of the COVID-associated pulmonary complications that develops even 6–8 weeks after initial infection with SARS-CoV-2, particularly in critically ill patients treated in intensive care units, is lung fibrosis, a condition that may also result from other causes such as cigarette smoking or long-term exposure to cigarette smoke. Although there is no data so far about protein citrullination level in COVID patients suffering from lung fibrosis, it was found that in a model of lung fibrosis induced by cadmium and carbon black (Cd/CB), two cigarette components providing a model for cigarette smoke, the level of citrullinated vimentin (Cit-vim) was significantly increased and was related with a TLR4-dependent activation of NF-κB and the production of active TGF-β1, CTGF (Connective Tissue Growth Factor) and IL-8 [157]. These ex vivo studies were confirmed in a mouse model in which control animals treated with Cit-vim developed TLR4-dependent lung tissue fibrosis, whereas *PAD2*^*−/−*^ mice, exposed to cadmium/carbon black did not generate high amounts of Cit-vim. This observation points at PAD2 as a promising potential factor in fibrosis treatment.

### Cancer

Studies in cancer cell lines demonstrated that the occurrence of histone citrullination, mediated by PAD4, on p53-regulated genes, including p21, CIP1, WAF1 and OKL38 repressed their transcriptional activation, leading to cell cycle arrest and apoptosis [158, 159]. These findings are in support of the notion that targeting PAD4 and other PAD enzymes may provide a novel strategy to inhibit tumor growth [160]. Immunohistochemistry and western blot detection demonstrated PAD4 overexpression in various tumor types, especially adenocarcinoma. In these samples, PAD4 overexpression was found to be associated with cytokeratin, a widespread tumor marker, in a citrullinated form that rendered it resistant to the digestion by caspases [161]. Elevated PAD4 levels were also observed by ELISA in the blood of patients with malignant tumors, but not in blood of patients with benign tumors [162]. Similarly, overexpression of PAD2 was observed in prostate cancer and correlated to histone H3 citrullination [163]. Administration of the pan-PAD inhibitor Cl-amidine synergized with the androgen receptor inhibitor enzalutamide to decrease prostate cancer cells proliferation in vitro and in xenografts [163].

Besides the direct promoting effect of histone/protein citrullination on cancer growth, a recent study on a cohort of 975 patients with cancer demonstrated that citrullinated histone H3, a marker of NETs and arterial thromboembolism, predicts the risk of all-cause mortality in patients with cancer [164].

### Thrombosis

A study by Fuchs et al. proved for the first time that NETs perfused with blood caused platelet adhesion, activation and aggregation demonstrating that NETs provide a structural platform and biological stimulus for thrombus formation [165]. PAD4, by promoting histone citrullination and chromatin decondensation, was identified to regulate both NETs formation and promote pathological thrombosis [166]. The overexpression of PAD4 was shown to promote, in an in vitro system, chromatin decondensation and formation of NET-like structures in cell types that do not undergo this form of cell death [167]. That PAD4 is necessary to mediate NETs formation was demonstrated by gene targeting of PAD4. Neutrophils derived from PAD4 knock-out mice were unable to generate NETs upon exposure to bacteria or stimulation with chemokines [168], and PAD4 knock-out animals were more susceptible to bacterial infection [168]. The link between PAD4 activity, NETs formation and induction of thromboembolism has been demonstrated in PAD4 knock-out mice subjected to a stenosis model to promote deep vein thrombosis: upon intervention, < 10% of PAD4 knock-out animals produced a thrombus, as compared to 90% of the control wild type animals [169].

## Diagnostic and therapeutic potential of citrullination

Recent years brought significant progress into the understanding of the role of the citrullination process in immune disorders. The growing body of evidence highlighting the importance of citrullination, and the identification of autoantibodies directed to the citrullinated peptides or proteins (ACPAs) has significantly broadened our understanding, and clinical characterization, of many immune disorders. The number of the ongoing clinical studies targeting PADs and the citrullination process is, however, currently limited to a few. The most advanced studies concern rheumatoid arthritis, and include diagnostic tests (Table 5) as well as interventional trials (Table 6).

**Table 5:** Diagnostic studies based on citrullination in the immune disorders

Analyzed parameter	Disease	Clinical trial registration number	Phase of the study
Subtypes of ACPA	Rheumatoid arthritis	NCT03832374	Descriptive study
ACPA antibody	Rheumatoid arthritis	NCT03663829	Non-interventional study
Anti-MCV Ab and anti-CCP Ab	Rheumatoid arthritis	NCT01078597 NCT03265236 NCT03224377	Diagnostic test

The clinical trial registration number from www.clinicaltrials.gov is provided. *Anti-CCP Ab* anti-cyclic-citrullinated peptide antibody; *Anti-MCV Ab* anti-mutated-citrullinated vimentin antibody

**Table 6:** Targeting autoimmune diseases using ACPAs

Drug	Disease	Clinical trial registration number	Phase of the study
Abatacept, Methotrexate	Rheumatoid arthritis, rheumatic diseases	NCT03492658	Interventional trial, Phase 4
Enbrel	Psoriatic arthritis	NCT04428502	Observational trial
Enbrel	Rheumatoid arthritis	NCT04428424	Observational trial

The clinical trial registration number from www.clinicaltrials.gov is provided

The non-interventional studies aim at the identification and quantification of the prevalence of anti-citrullinated proteins antibodies directed against different citrullinated antigens, including collagen, fibrinogen, filaggrin or vimentin, to evaluate their diagnostic and prognostic value in rheumatoid arthritis progression (Table 5, NCT01078597, NCT03265236, NCT03224377). Monitoring of anti-cyclic citrullinated peptide (anti-CCP) antibodies and anti-mutated citrullinated vimentin (anti-MCV) antibodies titers in RA, brought the conclusion that anti-CCP antibodies have a better diagnostic power than anti-MCV antibodies. However, anti-MCV antibodies have a higher sensitivity as compared to anti-CCP antibodies. A significant correlation has been found between anti-MCV antibodies titers and both the severity of RA and the disease-activity score (DAS28). Anti-MCV-positive patients presented lower reduction in disease activity and an increased number of swollen joints. This finding allowed concluding that anti-MCV antibodies titers may work as an indicator correlating the disease activity and patient outcome [170]. Also, an attempt was made to characterize the etiology of rheumatoid arthritis from lung level (NCT03832374), as it was found that diffuse interstitial pneumopathy and bronchial dysfunctions occur in one-third of RA patients, and that some subtypes of ACPAs are preferentially present in the lungs [171]. This study (NCT03832374), still ongoing, addresses a very important question regarding the subtype(s) of ACPAs with a preferential tropism for the lung, that might be responsible for the infection within parenchyma and pulmonary airways, and the overall increased morbidity and mortality among RA patients with lung co-morbidities.

Likewise, an observational study was performed to assess the impact of ACPA in RA participants who were treated with the immunosuppressive drug Abatacept or the tumur necrosis factor inhibitors (TNFis): adalimumab, certolizumab, etanercept or golimumab (NCT03663829). A total of 2052 patients were included of which 1415 were in the TNFis cohort (*n* = 1053 ACPA positive) and 637 in the Abatacept cohort (*n* = 445 ACPA positive). The baseline ACPA score was associated with an improvement in clinical response using a measure of clinical disease-activity index at 12 months for Abatacept therapy, but not for TNFis therapy [172].

As of today, most of the interventional trials are at an early clinical testing phase (Table 6). The most advanced clinical studies concern investigation of the effect of combination therapy of Abatacept and methotrexate on rheumatoid arthritis (NCT03492658). Abatacept (CTLA4-Ig) is a fusion protein, combining cytotoxic T cell antigen 4 (CTLA4) and IgG_1_ Fc_1_, dedicated to modulate the T cell co-stimulatory signal. Abatacept works as an immune checkpoint and downregulates immune responses mediated through the CD28–CD80/86 pathway. The dose ranging pilot study of patients with active RA, treated with both Abatacept and LEA29Y (Belatacept, a CTLA4-Ig variant), showed dose dependent reductions in the clinical manifestations of disease [173]. Following the pilot studies, an investigation of combined therapy with Abatacept and methotrexate, an immune system suppressant, is now ongoing for the determination of its effectiveness on RA phenotype, transcriptional profile of the disease, B cell receptor usage and functional parameters of circulating B cells expressing ACPA (NCT03492658). Patients will be randomized to treatment with (i) methotrexate monotherapy (10–25 mg once weekly) reference group or a (ii) combination therapy of methotrexate (10–25 mg once weekly) and Abatacept (125 mg subcutaneously once weekly) for 6 months, followed by methotrexate monotherapy (10–25 mg once weekly) in both groups for another 6 months. Phase II (NCT00254293) [174] and III (NCT00048568) of the trial gave promising results and showed that long-term treatment with Abatacept provided sustained efficacy with high RA patient retention [175].

Other ongoing clinical trials addressing the study of citrullination process are aiming to evaluate the effect of Enbrel treatment with regards to anti-cyclic citrullinated peptide in psoriatic arthritis activity (NCT04428502) and the impact of rheumatoid factor and anti-cyclic citrullinated peptide in rheumatoid arthritis (NCT04428424). Enbrel, also known as Etanercept, is a TNF-α inhibitor used to treat autoimmune diseases. In previous studies Enbrel efficacy was compared with disease modifying antirheumatic drugs (DMARDs) commonly used in patients with rheumatoid arthritis. It was found that Enbrel leads to a much greater decrease of the serum levels of anti-CCP and RF in rheumatoid arthritis than DMARDs alone. This finding was compatible with a reduction in clinical disease activity [176], Ann Rheum Dis 2006 Jan;65(1):35–9). Moreover, to evaluate the efficacy and safety of Etanercept, the drug was given over a 2-years period (25 mg twice per week) to patients suffering from different autoimmune disorders, including: ankylosing spondylitis (9 patients), juvenile rheumatoid arthritis (4 patients), rheumatoid arthritis (57 patients) and psoriatic arthritis (6 patients). This study confirmed the safety of Etanercept administration up to two years. Also, declined levels anti-CCP antibody and rheumatoid factor observed in this study, lead to the conclusion that a significant clinical response in different subclasses of arthritis patients can be achieved by Etanercept [177]. Further clinical studies on Etanercept and Abatacept addressing their anti-inflammatory potential will be necessary allow a full validation of their clinical value and, in the affirmative, a progression towards their routine clinical use.

As outlined above, the clinical studies addressing citrullination are focused around citrullinated protein antibodies as a diagnostic element to assess disease progression. However, a recent preclinical report delivered by Chirivi and co-workers provides evidence that ACPAs can also potentially be used as therapeutics for neutrophil-mediated inflammatory diseases such as inflammatory arthritis, pulmonary fibrosis, inflammatory bowel disease and sepsis [111]. Using these therapeutic ACPAs (tACPAs), particularly against citrullinated histones H2A and H4, it was found that in mice suffering from chronic arthritis these antibodies suppressed NET release and possibly initiated NET uptake by macrophages, resulting in reduced tissue damage in the joints [111].

## Conclusions and future perspectives

Recent basic, preclinical and clinical research in the early diagnosis of autoimmune disease, particularly arthritis, has shown that conversion of selected arginine residues to citrulline and the immune response to citrullinated proteins are key elements of recognition and better understanding the etiology of these disorders. It was shown that ACPAs are also directly involved in the pathogenesis of autoimmune disorders at several levels through (i) ligation to Fc receptors [178], (ii) modification of the function of monocytes [179], (iii) differentiation of osteoclasts [128] and (iv) formation of extracellular DNA traps [6]. Recent research has also shown that there is a potential link between the histone specific ACPAs (directed to citH2A and citH4) and NETs that deserves to be investigated and may lead to promising new strategies in treatment of autoimmune diseases [180].

It is now becoming evident that further studies on the effects of protein citrullination, biochemistry of citrullinating enzymes and modification of cellular proteins, and also on generation of ACPAs, will significantly help in understanding the metabolic processes as well as pathophysiology of multiple disorders, not only of immune/autoimmune origin, but also metabolic disease and cancer. A deeper look into the “citrullinome” and the cross-talk between citrullination and other modified residues may bring the development of novel drugs acting against disorders associated with aberrant citrullination.

## Data Availability

Not applicable.
